# Orienting Gaze Toward a Visual Target: Neurophysiological Synthesis with Epistemological Considerations

**DOI:** 10.3390/vision9010006

**Published:** 2025-01-14

**Authors:** Laurent Goffart

**Affiliations:** Centre Gilles Gaston Granger, UMR 7304 Centre National de la Recherche Scientifique, Aix Marseille Université, 13621 Aix-en-Provence, France; laurent.goffart@cnrs.fr

**Keywords:** poly-equilibrium, saccade, pursuit, fixation, neurophysiology, neuro-ophthalmology, monkey, cat, kinematics, dynamics, model, space, noise

## Abstract

The appearance of an object triggers an orienting gaze movement toward its location. The movement consists of a rapid rotation of the eyes, the saccade, which is accompanied by a head rotation if the target eccentricity exceeds the oculomotor range and by a slow eye movement if the target moves. Completing a previous report, we explain the numerous points that lead to questioning the validity of a one-to-one correspondence relation between measured physical values of gaze or head orientation and neuronal activity. Comparing the sole kinematic (or dynamic) numerical values with neurophysiological recordings carries the risk of believing that the activity of central neurons directly encodes gaze or head physical orientation rather than mediating changes in extraocular and neck muscle contraction, not to mention possible changes happening elsewhere (in posture, in the autonomous nervous system and more centrally). Rather than reducing mismatches between extrinsic physical parameters (such as position or velocity errors), eye and head movements are behavioral expressions of intrinsic processes that restore a poly-equilibrium, i.e., balances of activities opposing antagonistic visuomotor channels. Past results obtained in cats and monkeys left a treasure of data allowing a synthesis, which illustrates the formidable complexity underlying the small changes in the orientations of the eyes and head. The aim of this synthesis is to serve as a new guide for further investigations or for comparison with other species.

## 1. Introduction

Among the numerous relations that an entire animal establishes with its environment, the orienting movement was once proposed to underlie this interrogative process which “in Man has strongly developed in this highest form which is curiosity—the parent of that scientific method by which we hope one day to achieve a correct orientation in the knowledge of the world around us” [1]. This view remains popular in the domain of contemporary cognitive neuroscience wherein orienting is a module underlying exploratory behavior, gathering sensory signals, transforming them into information and storing them in some sort of internal copy of the external world.

In this article, we shall take a different point of view, avoiding teleological and cognitivist arguments as much as possible. We shall focus on the orienting movements of the eyes and head toward the location of an object. We shall explain how these two movements are behavioral outcomes of intrinsic brain processes, which are blind to the physical and informational relations occurring at the macroscopic level, that is, between the animal organism and objects in the external world. We shall start from the standpoint that the inner milieu consists of multiple equilibria maintaining some kind of stability. Whenever asymmetrical changes disrupt it, intrinsic processes restore the homeostasis. This restoration involves a coordinated set of reactions measurable at the musculoskeletal, vascular and vegetative levels. Orienting movements of gaze, head or body toward a target are the overt, externally visible consequences of activities traveling through the sensorimotor neural networks during such restorations. Other changes are less visible, such as transient pupillary dilation or cardio-vascular changes (bradycardia associated with peripheral vasoconstriction and cephalic vasodilation) [2,3], but also postural adjustments [4,5,6]. Synaptic changes and the persistent traces left by repetition are internal consequences that are indirectly visible by longitudinal investigations. In the same way as the sandy soil conforms to the current of a river while determining at the same time its flow, the sensorimotor neuronal channels assimilate to some extent the regularity of activity flows while constituting at the same time the set of constraints for the on-going flows.

Before detailing the neurophysiological substrate of orienting eye and head movements, it is useful to recall that they do not constitute the only type of reaction to an external stimulus. Other types include distancing, avoidance and escape. Orienting differs from them by the radically opposite way in which it manifests spatially in relation to the stimulus. Contrary to them, it consists of directing the body segment that carries the teleceptive organs towards the location of the stimulus, be it detected or coveted. In this article, we shall focus on two physical characteristics of animal reactions toward a visual stimulus: their velocity and their accuracy. Indeed, the orienting response consists neither of a sliding movement nor of a sequence of small movements directed toward the location of a targeted object. For the vast majority of animals, it manifests itself rather by a sudden and extremely rapid movement of the mobile segment (body, head or eye) that carries the photosensitive epithelium. The movement (also called saccade) consists mostly in a rotation, the amplitude and direction of which are limited to a range sufficient to symmetrize back the functional neuronal image of the stimulus within the sensorimotor networks.

**Figure 1:**
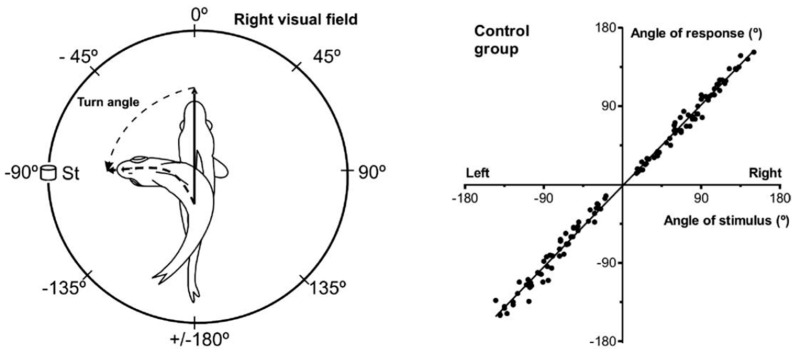
Orienting movement of a goldfish toward a small pellet of food dropping at different sites of a square tank. The rightmost panel shows the angle of response as a function of the angular eccentricity of the stimulus for a representative animal. Modified from [7] with the permission of the authors and Elsevier.

At the end of the orienting saccade, measuring the amplitude of the angular displacement performed by the mobile segment gives a set of numerical values that are compared to the target eccentricity. Figure 1 illustrates their relationship in a goldfish [7]. The amplitude of the body rotation is not constant; it increases with the angular eccentricity of the stimulus relative to the body, even in the absence of visual feedback. This relation has been documented in several other species: in frogs [8], salamanders [9], barn owls [10], cats [11], macaque monkeys [12,13] and human subjects [14,15,16]. Also observed in invertebrate animals such as insects (mantis: [17,18]; dragonfly: [19]) and crustaceans [20], it suggests that, beyond the diversity of photosensitive surfaces (retina or compound eyes), of muscle fibers or of their attachments—indirect or direct—to the mobile segment, the extension of physical space has somehow been embedded within the neural networks.

In cats and monkeys, the relation is not a fixed mapping between the topology of the photosensitive surface and a set of specific temporal commands changing the orientation of eyes and head. Experiments in these animals showed that this behavioral invariant is robust to changes in saccade trajectory induced by a brief electrical stimulation in some territories of the brainstem (cat: [21,22]; monkey: [23,24,25,26,27]) or cerebral cortex (monkey: [28,29]). However, when the perturbation is applied in some sites of the pontine reticular formation [24] or in the medio-posterior cerebellum [30,31], the tested monkey orients its gaze toward a location that is shifted with respect to the target location. The saccade misses the target by an error equal to the stimulation-induced change in eye position. This targeting error indicates and confirms that extraocular muscle proprioception does not participate in guiding saccades toward their target [32,33].

## 2. Fundamental and Epistemological Issues

In response to the same initial physical conditions, the amplitude of the response is not constant but variable within some limits. Beyond the multiplicity of channels by which retinal signals can reach the motor neurons, beyond also the time course of the persistence of signals, the amplitude variability is also due to the facts that objects in the external world are not points and that visual fixation does not involve the projection of their image onto one single light-sensing cell, but several ones. In some species, the photosensitive cells are grouped within the foveal pit. Whenever possible, we shall use the notion of “gaze” instead of the so-called “line” of sight (or visual “axis”) to denote the imaginary “tube” through which photons travel from the fixated object in the external world to the foveal photoreceptors.

Despite quasi-inevitable numerical differences, some degree of congruence must be considered between the location of a visual stimulus and the zone toward which gaze is directed. As mentioned above, this overlap is preserved even when the functioning of some key sensorimotor regions is perturbed. Before reporting these findings and discussing their implications, we shall address a few epistemological issues related to the risk of importing, within the domain of neurophysiological knowledge, notions whose biological plausibility remains uncertain or dubious, see also [34].

### 2.1. Distinguishing the Medium of Cerebral Activity from Physical Space

The “binocular fixation point” is defined as the intersection between two imaginary lines that characterize the direction of each visual axis while the “vergence angle” is the angle made by these two lines [35]. When that angle decreases, the eyes are said to converge and one speaks of convergence. When it increases, they are said to diverge (divergence). In the subject with no strabismus, when a saccade shifts gaze between two targets located along an iso-vergence curve, the spatial trajectory of the binocular fixation point follows neither the shortest physical path between the targets (the straight line) nor a path along the iso-vergence curve. Relative to the frontal plane, the binocular fixation point takes a path away from the iso-vergence curve followed by a return to it: a transient divergence happens during the saccade. The path traveled by gaze lies in a “geodesics” that does not belong to the zero-curvature space of Euclidean geometry. There is no force field in the external world that constrains gaze to take such a path. The path is intrinsically determined, caused by the neuronal networks upstream of the extraocular muscles.

As pointed out by Pellionisz and Llinas, “since within the brain there is no ‘instantaneous’ simultaneity agent comparable to the light, the classical usage of separate space and time coordinates is inapplicable in the case of describing the inner workings of the central nervous system” [36]. From a naive point of view, the timeless entity that geometers and physicists designate as “space”, the receptacle occupied by objects, might correspond to the medium within which flows of neuronal activity propagate, from the excitation of sensory cells by external objects to the time when the orienting reaction ends. However, the cerebral networks radically differ from the three-dimensional framework that assigns numerical values to positions and displacements of objects. They constitute a medium that is neither isotropic nor homogeneous, but composed of multiple and diverse channels. Each physical place captured by gaze is the outcome of multiple flows of activity through this medium. The divergence characterizing the afferent pathways first broadens the neural image of a small spot of light, an image then compressed by the convergence of activities towards the motor nerves. Transforming the recruitment of excitatory and inhibitory synapses and the firing rate of neurons to algebraic time functions engenders a continuum that is not in accordance with the remote and distributed conditions of action potential triggering [34].

The multiple sensorimotor channels cannot correspond to a neural representation (or correlate) of physical “space” for other reasons. The numerical values assigned to different physical target positions or movement endpoints are independent of each other. They are ordered and aggregate to each other within another continuum, which corresponds to an infinite number of simultaneous points (mathematical space). In the brain, there is no corresponding entity. The elements are neither compact nor rigid, and their number is much smaller than the number of objects present in the external environment of a subject. Activities in the brain corresponding to different physical locations are neither points nor lines. Those leading to the generation of movements towards nearby but different locations can involve common neurons. Indeed, neurons have extended and overlapping response fields [37]. The sharing of neuronal elements is such that two simultaneous visual targets can lead to a saccade (called averaging saccade), which directs gaze toward a location situated midway between the two targets or along the line connecting them [38,39].

Moreover, measuring a series of motor responses to targets located at different places in the visual field is not equivalent to measuring the entire field. The measurement values obtain their status of simultaneity after their plotting on two- or three-dimensional graphs. Whatever corresponds to physical space or to objects in the brain, it is not permanent, but short-lived. Its persistence requires either the presence of objects in the external world or in working memory, which adds numerous contextual non-spatial values, past and present [40,41]. When we consider the accuracy and precision with which saccades indicate the location of a target, they depend considerably on its presence. When it is produced shortly after target appearance, the saccade is sufficiently accurate to confirm the presence of the object by a set of sensations, which are relatively always the same and characterize the foveation. Regarding the object location, the kind of “measurement” made by saccades is, so to speak, reliable. By contrast, when the saccade is made after a delay, even after a relatively short one, the “measurement” is no longer reliable [42,43,44,45,46]. Visual sensations can no longer confirm the presence of the object since it has disappeared while the change in saccade accuracy and precision make the movement-related sensations uncertain. Saccades are also strongly inaccurate (hypermetric) when they are made with the eyes closed [6,47]. The persistence of visual signals in the brain requires the presence of objects to create a “space”, in which case its internal representation (or neuronal copy) is either rapidly unreliable or unnecessary since it is already out there [48].

Finally, neural plasticity, the irreversibility of activities and their canalization argue for a fundamental difference between the cerebral medium and the Newtonian receptacle. Yet, the adaptation of movements to environmental constraints led some authors to propose that brain activity emulates internal models of the physical laws of motion. Unfortunately, they failed to explain how the diversity of animals and nervous systems is compatible with Newton’s three laws of motion. Leaving aside the principle according to which rest corresponds to a balance of forces that cancel each other out, they did not explain how brain activity could sustain the motion of a body segment at constant speed with muscles of finite length either. If the laws of mechanics are conventions [49,50] used to quantify the movement of objects in space, then it calls into question whether it is reasonable to assume their applicability to the brain sciences. The consistency and viscosity of the cerebral medium in which activities propagate are not as homogeneous as those of the environments (water, ground and air) in which the animal body can evolve. As for the eye movements, their study in untrained monkeys shows a tracking that is not as smooth as the continuous and uniform motion of a visual target. The visual tracking is discontinuous and saltatory, interrupted by catchup saccades [51,52,53], indicating that the streams of visuomotor activity do not spontaneously mimic the constant motion of the target. The fact that repeated practice is required to perform spatially and temporally accurate movements does not imply cerebralization of physical laws. It merely illustrates the flexibility of the brain’s functioning to perform accurate movements, perhaps by eliminating irrelevant or distracting stimuli.

Refusing to import notions pertaining to the kinematic or kinetic description of movements onto the activity of neurons does not lead the neurophysiology of eye–head movements to a dead end. We shall see that it actually offers the opportunity to make a fresh start and to investigate how the brain works differently from the way that has been promoted during recent decades.

### 2.2. The Spatiotemporal Transformation

A fundamental challenge in the brain sciences is to identify and extract processes from the complex networks of cells that are often polyvalent. Between the retinal ganglion cells and the extraocular and neck muscle fibers, the action potentials emitted by neurons are involved in multiple tasks, making the delineation of processes far from being a trivial task. The mere excitation of a small group of ganglion cells in the retina by a stimulus as simple as a brief spot of light is transmitted to a multitude of postsynaptic neurons in several deep cerebral nuclei (e.g., lateral geniculate nuclei, superior colliculi, nuclei of the optic tract). The action potentials emitted by these neurons are then transmitted to more numerous postsynaptic cells distributed in multiple areas of the cerebral cortex. The spot of light elicits a neural activation that spreads out within the brain’s networks. Its expansion is associated with a temporal persistence insofar as the duration of elicited activities outlasts that of the stimulus. This maintained activity is necessary for changing the firing rate of specific groups of motor neurons and for driving the contraction of extraocular and neck muscles until gaze is oriented toward the location of the spot of light, sometimes a long time after its disappearance.

The process by which different loci and more distant stimuli in the visual field (i.e., more eccentric loci of retinal activity relative to the fovea) lead to different and longer motoneuronal discharges has been called “spatiotemporal transformation”. Such a transformation is not limited to orienting movements toward a visual target. It characterizes all movements directed toward events that are spatially delimited, such as an object in the visual field or a point stimulation on the surface of the skin, as well as toward events whose physical location is more diffuse such as sounds or screams. In all cases, a premature arrest of motoneuronal activities will make the movement hypometric, i.e., falling short of the target location.

Initially, the dysmetria of movements made by patients suffering from cerebellar dysfunction led to the proposal that the cerebellum performed this spatiotemporal transformation [54,55]. The afferent signals from retinotopically organized maps such as the one found in the superior colliculi were thought to be converted into a specific temporal pattern of motor activity (see Figure 10 in [56]). However, the observation of accurate saccades in patients suffering from spinocerebellar degeneration, despite dramatic slowing and increased duration, led to this hypothesis being abandoned [57]. The conjecture was then proposed that saccade duration be determined by the work of a negative feedback loop [57,58,59]. The premotor neurons would be driven by a motor error command that results from the continuous comparison between two signals: a reference signal specifying the goal or desired state (desired eye orientation or desired change in eye orientation), and a feedback signal estimating the current state (current eye orientation or actual change in eye orientation). As long as a mismatch persists between these two signals, the motor error command drives the premotor neurons until the estimated current state matches the desired state. In this theoretical framework, there is no signal specifying the duration of saccades; it merely corresponds to the time taken by the feedback signal to zero-out the motor error command. Saccade inaccuracy results either from an altered reference signal or from an impaired feedback signal. Transient changes in the trajectory of saccades do not affect their accuracy as long as they do not alter the signal that specifies the saccade goal, i.e., the amplitude and direction required to foveate the target [21,22,23,24,25,26,27,28,29].

For several years, the negative feedback loop remained the simplest and widely shared hypothesis to account for the spatiotemporal transformation. The reference signal was proposed to be conveyed by the population of active neurons in the intermediate and deep layers of the superior colliculus [60,61,62,63] while the comparison with feedback signals took place downstream in the reticular formation, under the influence of neurons in the medio-posterior cerebellum [30].

**Figure 2:**
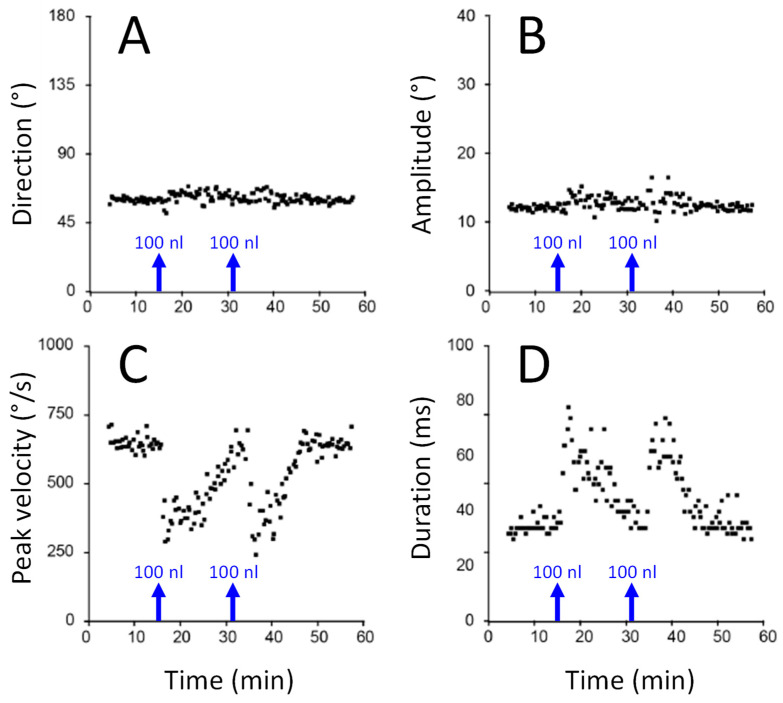
Effects of suppressing the activity of a small set of collicular neurons on saccades toward a visual target. A small volume of lidocaine (100 nl) was injected two times inside the population of active neurons while a monkey made saccades toward a target. Immediately after each injection (blue arrow), the peak velocity of saccades was reduced (**C**) while their duration was lengthened (**D**). In comparison with these changes in velocity, the direction (**A**) and amplitude (**B**) of saccades were barely affected. Courtesy of Dr David L. Sparks, modified with his permission.

Figure 2 illustrates how the direction (A) and amplitude (B) of saccades toward a visual target are preserved in spite of their dramatic slowing when the activity of some neurons in the deep superior colliculus is suppressed by local injection of a blocker of sodium channels [63]. After each injection of a small amount of lidocaine, the maximum velocity of saccades is considerably reduced (C) while their duration is lengthened (D) by an amount that appears proportional to the amount of velocity reduction. In this experiment, the injection did not silence all collicular neurons, but only those that fired maximally for saccades having a particular direction and amplitude. Saccades with amplitudes and directions that were close to this preferred vector were inaccurate [61]. In addition to demonstrating that saccade metrics was specified by the population of active neurons in the superior colliculus [60], this study brought evidence for a causal link between collicular activity and saccade velocity and for a feedback control of saccade amplitude and direction (see also [64]).

Around the same time, works on head-unrestrained cats led to the proposal that the comparison between the reference and feedback signals was performed within the superior colliculus (SC) itself, by a caudo-rostral migration of activity [65,66]. The change in the locus of collicular activity would switch from the recruitment of saccade-related neurons to the recruitment of fixation-related cells. The former neurons would be distributed across the entire extent of the SC whereas the latter would be concentrated in its rostral part. The saccade would stop as the “fixation” cells resume their sustained presaccadic firing rate, which in turn, would excite cells in the nucleus raphe interpositus (RIP) of the medial pontine reticular formation. Therein, neurons called omnidirectional pause neurons (OPNs) exhibit a sustained spontaneous firing rate and pause during each saccade, regardless of its amplitude and direction. Their sustained activity was proposed to inhibit premotor burst neurons and pause to trigger a saccade.

However, subsequent studies disconfirmed the migration of activity across the SC during saccades [67,68,69,70]; but see [71]. This lack of reproducibility led to abandoning the conjecture (called “moving hill” hypothesis) of a caudo-rostral migration of collicular activity (exception in [72]). Later, confirming observations reported in an earlier study [73], neurons in the rostral SC were shown to emit a burst of action potentials during miniature saccades [74,75], extending the saccade-related function to this part of the SC, as a microstimulation study also suggested [26]. Indeed, contrary to electrical microstimulation of RIP, which only decelerates and interrupts visual saccades, microstimulation of the rostral SC deviates their trajectory toward the contralateral side. The absence of hypermetria after pharmacological inactivation of the rostral SC [76,77] further called into question its role in terminating saccades whereas the reduced rate of microsaccades during visual fixation [74,77] confirmed its involvement in the generation of miniature saccades [73,78]. Finally, the lack of impairment in either the latency or the accuracy of saccades after RIP lesions [79,80] questioned the involvement of OPNs in fixation and saccade termination. It also cast doubt on the idea that silencing their activity disinhibits premotor burst neurons and allows their excitation by descending SC commands [81]. Indeed, suggestions were also made (i) that OPNs’ sustained activity prevents the visual response of SC neurons from driving premotor burst neurons and (ii) that saccades with very short latencies (also called express saccades) were driven by that visual response [82,83]. However, the demonstration of a loose coupling between the saccade onset and the visual (rather than motor) response of SC neurons [84,85] contradicted these suggestions. As for saccades with regular latencies [86], the onset of express saccades is tightly coupled with the onset of motor burst of SC neurons [84]. The variability of saccade onsets likely depends upon the synchronies of action potentials impinging upon the premotor burst neurons: weaker synchrony yielding scattered and longer reaction times. As explained elsewhere [52], simultaneous excitatory action potentials from an assembly of neurons are more efficient in exciting their postsynaptic targets than temporally scattered action potentials.

Altogether, these numerous studies and others led to the realization that the spatiotemporal transformation is not explicitly performed by distinct groups of neurons, which neurophysiologists ought to search for and identify within the brain. Instead, the transformation would be supported by activities of populations of neurons that are massively interconnected and distributed over several neural territories [87]. We shall see in the next section how this distributed character also holds for the cerebral control of gaze direction during visual fixation.

### 2.3. Gaze Direction as a Poly-Equilibrium

Several lines of evidence led to the proposal of an alternative hypothesis to the “fixation–saccade” dichotomy [88,89,90,91]. First, the absence of fixation instability with lesions or during pharmacological inactivation of RIP [79,80] questions the necessary involvement of OPNs in maintaining stable gaze. Second, numerous studies performed in awake animals report cases in which the animal does not accurately direct its gaze toward a visual target during fixation or does not correctly orient its head toward a food target. During unilateral inactivation of the caudal fastigial nucleus (cFN) with muscimol (Gaba-A agonist), cats direct their gaze and mouth toward a location that is offset with respect to the physical target [11]. A comparable eccentric fixation happens in head-restrained monkeys during unilateral pharmacological inactivation of cFN. Therein, the local and unilateral injection of muscimol leads to an ipsilesional fixation offset [92,93,94,95] whereas the injection of a Gaba-A antagonist (bicuculline) yields a contralesional offset [96]. The muscimol-induced fixation offset is not a defect in positioning the eyes in the orbit, but a gaze-related disorder because its size is quasi-identical when comparing cases in which visual fixation is made with the head restrained versus unrestrained [97]. Moreover, when the head is free to move, a large ipsilesional deviation affects its orientation and the deviation of the eyes in their orbit is contralesional [97]. Finally, a weakening of stability accompanies the fixation offset insofar as monkeys have difficulty in maintaining their gaze near the central target when other behaviorally relevant targets appear in the peripheral visual field: the animals make irrepressible saccades toward their location during delayed oculomotor tasks (unpublished observations; see the increased rate of no-go errors in [98]).

Anatomical studies report that cFN neurons project neither to the nucleus prepositus hypoglossi nor to the medial vestibular nucleus [99], nuclei in which tonic eye position neurons are located [100]. However, cFN neurons project to the rostral part of both superior colliculi [101]; an anatomical projection consistent with the gaze-related disorder during fixation after cFN inactivation. Thus, during unilateral pharmacological perturbation (inactivation or disinhibition), asymmetrical and sustained activities from neurons in the left and right cFNs would affect the balance of bilateral rostral SC activity whenever gaze is directed toward a target in the central visual field. This conjecture is confirmed by the consequence of unilaterally inactivating small sectors in the rostral SC. After local injection of small amounts of muscimol, monkeys exhibit an offset when they fixate a target, regardless of whether it is static [77] or moving [102]. They also make irrepressible saccades toward the peripheral target during a memory-guided saccade task [76]. Transmitted to precerebellar pontine nuclei (dorsolateral pontine nucleus and nucleus reticularis tegmenti pontis) [99,103,104,105,106], asymmetrical and sustained activities between the left and right cFNs and rostral SCs would cause eccentric fixation of a static [92,93,94,95,96] or moving [102,107,108] target through a ponto-paraflocculo-vestibular route. Concerning the ipsilesional deviation of the head during unilateral cFN inactivation [11,97], it may result from asymmetrical sustained input from fastigial nuclei to the group of reticulospinal neurons innervating the neck motor neurons [109,110,111].

Offsets while directing the eyes toward the location of a static visual target have also been reported in monkeys after unilateral lesions [112] of frontal eye fields or during unilateral inactivation of a small part of them [113]. By contrast, unilateral lesion of area LIP, another major source of cortical input to SC [114,115], does not lead to a fixation offset [116,117,118]. A spontaneous nystagmus in the dark has been reported during the first week after unilateral ablation of area 7 of the parietal cortex [119]. Slow eye movements are directed toward the contralesional side. Clinical studies of patients suffering from an acute hemisphere stroke report a conjugate deviation of the eyes [120,121] and head [122] toward the lesioned side, even when subjects explore a visual scene. However, to our knowledge, their fixational eye movements have not been studied. By contrast, an asymmetric distribution of fixational saccade amplitudes toward the blind visual field has been shown in patients suffering from homonymous hemianopia; some subjects also exhibit eccentric fixation [123].

Fixation offsets are not restricted to lesional cases. An upward offset is observed when macaque monkeys look at a small visual target in the dark [124,125,126]. Asymmetrical representations of the upper and lower visual fields in these primates [127,128] can account for the offset as well as for the upward bias of horizontal saccades [126]. While the population of active neurons is immune to this asymmetry in darkness, it may be different when a visual target is presented against an illuminated background. More cells sensitive to the lower visual field may indeed be recruited and counteract the upward bias caused by the asymmetrical representation of the upper and lower visual fields. In darkness, the absence of their recruitment would then account for the upward bias of horizontal saccades. Further investigations are still required to test whether bilateral activity from neurons in the cerebellar interpositus nuclei adjusts this asymmetry [126].

**Figure 3:**
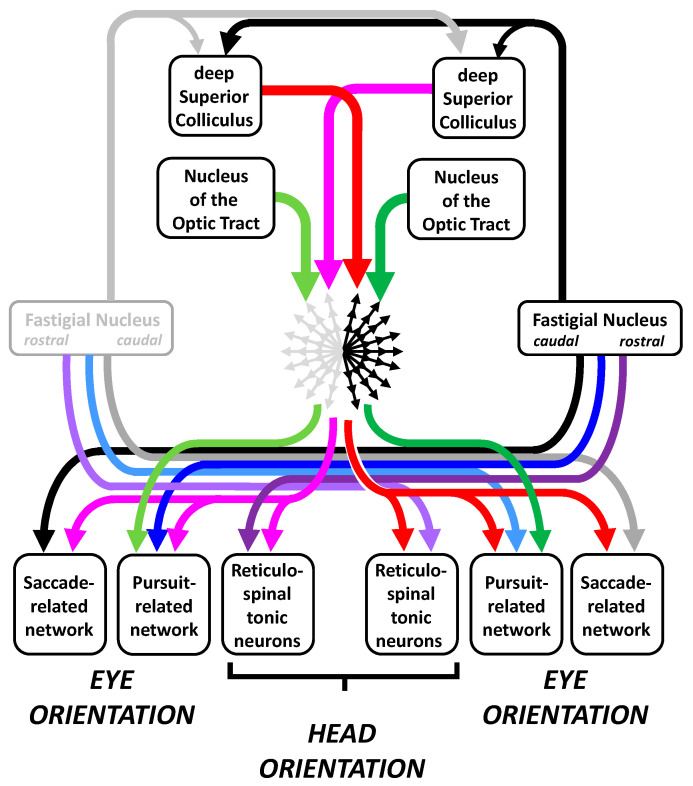
Poly-equilibrium hypothesis: A saccade or a slow eye movement is not initiated when the visuo-oculomotor system is at equilibrium, i.e., when opposing commands (issued, for instance, by the left and right superior colliculi or by the left and right nuclei of the optic tract) counterbalance each other. For generating saccadic and pursuit eye movements, symmetry breaking involves different groups of neurons. For saccades, premotor neurons are located in the pontomedullary reticular formation whereas, for slow eye movements, they are located in the vestibular nuclei. Bilateral fastigial activity also contributes to neck muscle tone, which specifies the horizontal orientation (yaw) of the head through fastigio-reticular projections. Different colors are used to distinguish the crossed and uncrossed channels. Read text for explanations.

Altogether, these results led to revision of the view of fixation as a process inhibiting the generation of saccades [76,88,89,90] and to a proposal on the conjecture of fixation as an equilibrium [77,129,130], later revised to a “poly-equilibrium” [131]. According to this theory (Figure 3), a saccade or a slow eye movement is not initiated as long as the visuo-oculomotor system is in a mode where opposing commands counterbalance each other. For generating saccadic and pursuit eye movements, symmetry breaking involves different groups of neurons in the left and right parts of the brainstem. Saccades involve the recruitment of premotor burst neurons located in the pontomedullary reticular formation whereas slow eye movements involve the recruitment of premotor neurons in the vestibular nuclei. Prior to generating a saccadic or slow eye movement, the target-related signals do not travel toward the motor neurons across a vacuous medium. They travel through a large network of cells that already evince a spontaneous sustained firing rate, which maintains the stationarity of the eyes and head. This set of tonic premotor activities, once called “neural integrator”, altogether forms an equilibrium of commands that counterbalance each other. Under normal conditions, gaze direction is relatively stable during visual fixation, occasionally interrupted by saccadic intrusions or by ocular drifts.

The notion of poly-equilibrium is more appropriate for naming this balanced activity because multiple equilibria participate. It involves action potentials conveyed through (i) the visuomotor channels that lead to the generation of saccades, (ii) the visuomotor channels that lead to the generation of slow eye movements, (iii) those yielding the near response (accommodation and vergence) and (iv) those holding the head upright [131]. The change in head orientation during unilateral lesion of the cFN [11,97] or the SC [132] indicates that bilateral activity also contributes to the muscle tone that specifies the static orientation of the head in the earth horizontal (yaw) plane. Sustained activities are distributed within different groups of neurons in the left and right parts of the reticular formation (including pontine long-lead burst neurons and pursuit-related neurons), cerebellum, deep superior colliculi and cerebral cortex. Concerning the deep superior colliculi, the activity involves their rostral pole but it is not restricted to them. It extends more caudally, even during steady fixation. Indeed, during the gap interval, saccade-related neurons in both SCs fire, even those located at sites that are sensitive to target eccentricities of 8 deg (see Figure 4 in [84]). Any suppression of activities within the set of commands participating in this poly-equilibrium alters the direction of gaze while fixating a target if it is not counterbalanced.

### 2.4. Movement Onset as a Kind of Symmetry Breaking

Considering static gaze direction as a poly-equilibrium leads to viewing unbalanced activities as the cause triggering eye movements, saccadic or slow. Unbalanced activities between the left and right superior colliculi promote the generation of saccades toward the side opposite the most active SC. By contrast, unbalanced activities between the left and right nuclei of the optic tract promote a slow eye movement toward the side of the most active nucleus (Figure 3). This laterality contrast is indicated by the contralateral direction of saccades evoked by electrical stimulation in the SC [133,134,135,136] and the ipsilateral direction of slow eye movements evoked by stimulation of the nucleus of the optic tract [137,138]. It is also indicated by unit recording studies. The firing rate of neurons in the nucleus of the optic tract increases during ipsilateral pursuit and decreases during contralateral movements [138,139,140] whereas SC neurons burst during contralateral saccades [60,133,141].

Slow eye movements result from action potentials transmitted by neurons in the vestibular nuclei and prepositus hypoglossi to the motor neurons innervating the extraocular muscle fibers [142,143,144,145]. In darkness or in some pathological cases, unbalanced inputs from the nuclei of the optic tract or from the floccular complex lead to a slow eye movement. The slow eye movement is then interrupted by a rapid eye movement in the opposite direction, as in patients suffering from downbeat nystagmus [146,147], after unilateral lesion or inactivation of NOT [129,148,149] or after lesion of area 7 of the cerebral cortex [119]. The sequence of slow and rapid eye movements making the nystagmus illustrates the segregation between the pursuit-related and saccade-related circuits. Thus, in response to a small moving spot, the increased firing rate of neurons in the ipsilateral nucleus of the optic tract moves the eyes slowly (pursuit) whereas the burst of neurons in the contralateral SC drives catchup saccades. While tracking a target moving horizontally, interactions between the pursuit and saccade-related circuits take place in the prepositus hypoglossi and medial vestibular nuclei, toward which premotor burst neurons [150] and neurons of the nucleus of the optic tract project [151,152]. They can also take place in the SC through inhibitory input from NOT to SC [152,153]. We shall discuss the synergy of saccadic and slow eye movements in more detail in another section.

### 2.5. The Wanderings of the “Brain Machine”

At the time when bioengineering approaches started to guide the neurophysiological inquiry of eye movements with systems analysis techniques, David A. Robinson already warned that “block diagrams of oculomotor organization serve as a compact description of system behavior but seldom have much bearing on the way in which the real system, composed of nerve and muscle, actually operates. The models thus do not contribute much to the neurophysiology (or neurology) of eye movements and incur the danger of suggesting that there actually are segregated portions of the nervous system which perform the differentiation, integration and other operations indicated in the boxes of the diagrams” [154]. Although these concepts are useful to the search for “a continuous chain of intelligible causes and effects and for arriving at a holistic view of problems, they do not exist as separable entities in computational neural networks”, which are “a much closer analogue of biological networks than the classical cybernetic description” [155]. Neuroanatomical and neurophysiological studies reveal indeed functional properties and connectivity that are not visible in cybernetic diagrams. Establishing correspondences between cybernetic diagrams and the brain is analogous to comparing an automaton to a living animal. For explaining variable sensorimotor performance, such a comparison led some authors to search for various sources of signal “noise” within the brain activity rather than for differences between brains and machines. Contrary to a machine, recruiting the same group of photosensitive cells does not lead to the same response, because of the multiplicity of visuomotor channels and the different modes of signal transmission [34]. As explained above, between the sensory “device” and the motor “plant”, there is not a single transmission channel, but a plexus of multiple channels, which first diverge (from the optic nerves) and then converge (to the motor nerves). The variability of movements may not be caused by noisy signals in the brain; it may be the mere consequence of the epistemological fact that the complete set of causes is not known.

In spite of evidence for reliable saccade motor commands [156], some authors posited their variability [157] and even imagined various kinds of signal noise (e.g., sensory noise, planning noise, premotor noise, motor noise, etc.) to explain cerebellar control of saccade precision [158]. Between the retinal activity and the ultimate muscle fiber twitch, the distribution of action potentials within the brain is inevitably variable between movements made toward the same target. However, the search for noisy signals must not ignore the importance of starting eye position in determining the locus of activity on the retina and the possibility of different functional connectivity between different subjects. Moreover, even though two brief and spatially distinct spots of light excite different retinal regions, the resulting activity will likely involve common neuronal elements in the brain because of the divergent pattern of connectivity. Response fields of central neurons that overlap are the main evidence for the sharing of neuronal elements during visual stimulations that are spatially distinct. This situation does not lead to unreliable visuomotor transformation if “the precision or accuracy of a saccade results from the summation of the movement tendencies produced by the population of neurons rather than the discharge of a small number of finely tuned neurons” [60]. The effects of variable discharges of neurons is reduced by averaging the activity over a population of numerous neurons. Thus, multiple combinations of active neurons can lead to kinematically (or dynamically) identical eye movements, reducing further the strength of the correlation between the firing rate of a single neuron and movement kinematics (or dynamics) [34]. While point values are assigned to the target location and the place to which gaze is directed, a geometric segment (called error) to their difference and a curve to the movement trajectory, their brain correlates, streams of action potentials, are unmatched and incomparable.

## 3. Orienting Gaze Toward a Moving Target

Many objects in the world are moving and orienting movements are also made toward them. In primates, their appearance in the peripheral visual field elicits a saccade that brings the target image onto the foveae. Then, the foveation is maintained by slow pursuit eye movements interrupted by catchup saccades. Movement accuracy consists of directing gaze toward the location where the target is here and now, the target location at the saccade landing time. The temporal interval between the onset of retinal signals and the landing time led some authors to defend a view according to which the functioning brain would be endowed with so-called “predictive” capabilities. In this section, we shall explain that this inference is the consequence of conflating numerical values attributed to physical events with their physiological correspondence.

### 3.1. The Interceptive Saccade

Benefiting from high-resolution recording of eye movements, studies showed that human and non-human primates manage to direct their gaze toward the location of a small target moving at constant speed [159,160,161,162] with an accuracy that is quasi-similar to that of saccades toward a static target [163]. In monkeys, this ability to intercept a small moving target with the gaze seems to depend neither upon prior experience [51] nor upon the occurrence frequency of the path taken by the target [164]. It is preserved even when the saccade is perturbed prior to its onset by an unexpected change in eye position [165]. However, saccades toward a moving target are not always accurate. Accuracy is lost when the target does not move at constant speed: saccades to an accelerating target fall short of it whereas those to a decelerating target overshoot it [166].

The ability to bring the image of a moving target within the central visual field led some authors to suggest an ability to foresee (or “predict”) the location that the target will occupy at the time of saccade landing, i.e., in the near future. Indeed, because of the polysynaptic path between the retinal ganglion cells and the extraocular muscles, the signals that drive the firing rate of motoneurons cannot originate from the retinal site that the target is crossing presently. They originate from a site that was crossed several tens of milliseconds earlier. Accurate prediction would then require accurate estimation of the visuomotor transmission delay, which has no fixed value. It depends upon the number of active neurons, their firing rate and the simultaneity of presynaptic action potentials at the level of each relay [52]. As for saccades toward a static target [167,168,169], the latency of interceptive saccades may also depend upon the target contrast.

A study in non-human primates regarding saccades toward a transient target moving with a constant speed reports several saccades landing beyond the location where the target disappeared, but along the path that it would have taken if it had remained visible [166]. Because the monkeys had no prior experience with such visual targets (small and moving), the saccades cannot be guided by memory-related signals, but by signals that persist after target disappearance. The kinetic of the retinal streak (the time course of the target motion in the visual field) is taken into account because, for identical paths, the pattern of landing positions depends upon the rate of change in target speed. As mentioned earlier, saccades are accurate if the target moves at constant speed; they overshoot its current location if it decelerates and fall short if the target accelerates. It is not known whether accelerating and decelerating targets recruit different neuronal networks or whether the different landing positions result from different spatiotemporal patterns of activity within the same plexus of neurons. As for curved saccades made in response to a target that steps from one location to another (double step saccades [170]), the recruitment of the same plexus for accelerating and decelerating targets would be further evidence for the importance of considering the distribution of activity within the neuronal networks for the causal determination of behavioral responses. Indeed, although important, the knowledge of anatomical connectivity is not sufficient to understand the neural control of movements. Lesion studies teach us that static and moving targets involve different sets of neurons: impairment of some brain regions can affect saccades toward a moving target without altering those toward a static target [171,172,173].

### 3.2. The Postsaccadic Slow Eye Movement (Pursuit)

A slow eye movement (called pursuit eye movement) follows the interceptive saccade toward a moving target. Studies in monkeys revealed that its speed does not spontaneously match with the target speed and that it increases with the animal’s training and prior experience [51,52,53]. Comparable observations were made in children [174,175]. In response to a continuous motion, naive monkeys and children track a target with a succession of catchup saccades interspersed by intervals during which gaze direction lags behind the target, with a gaze–target distance increasing until the next catchup saccade.

Clinical studies in human subjects [176,177,178,179,180] and lesion studies in macaques [107,181,182,183,184] indicate that saccadic and pursuit eye movements recruit different sets of neurons. Indeed, some lesions impair slow pursuit eye movements without altering saccades and vice versa. The segregation of saccade-related and pursuit-related networks is confirmed by: (i) developmental studies reporting different maturation speeds of saccadic and pursuit performances [174,185,186,187,188], (ii) separate adaptive adjustments of saccadic and pursuit eye movements [189,190,191], (iii) different consequences on postural stability [192], (iv) imaging studies in healthy human subjects [193,194] and (v) the electrophysiological identification of saccade-related and pursuit-related neurons in distinct regions of the pontine tegmentum [195,196,197] and cerebral cortex [198,199,200,201,202,203,204]. In the cerebral cortex, the saccade-related subregions are preferentially interconnected with each other, and likewise for the pursuit-related subregions [204]. In summary, from the cerebral cortex to the reticular formation, the networks involved in the generation of pursuit and saccadic eye movements are relatively independent and parallel.

This parallelism contrasts with the claim that a “single sensorimotor process” drives the generation of both movement categories [205,206], a conjecture that is mostly based on behavioral observations and on a mistaken interpretation of neurophysiological observations. First, the fact that neurons in the rostral SC modulate their firing rate during both miniature saccades and pursuit [207,208,209] is the neural substrate of looking straight ahead, i.e., moving gaze neither rightward nor leftward, neither upward nor downward, but promoting movements “in all directions at the same time” (Figure 3). As explained above, bilateral rostral SC activity maintains the poly-equilibrium necessary to maintain the target image within the central visual field, regardless of whether it is static or moving, actual or virtual. Second, it is premature to conclude an involvement of OPNs in pursuit eye movements because their electrical microstimulation slows them [210,211]. The slowing could result from inhibiting motor neurons by the retrograde excitation of inhibitory burst neurons [91] if this connection is confirmed in monkeys (see [212] for lack of evidence). Third, the observation that subthreshold electrical microstimulation in vermal lobules Vic–VII perturbs both saccadic and pursuit eye movement [213] remains compatible with a segregation of saccade-related and pursuit-related neurons in the cerebellar vermis. Therein, Purkinje cells firing during both saccades and pursuit are not numerous (approximately 10% [214]). Based on a large sample of neurons, another study reported that only 4% of saccade-related Purkinje cells modulated their firing rate during pursuit [215]. More recently, the direction tuning of Purkinje cells was found to differ between saccadic and pursuit eye movements, suggesting an independent processing of their action potentials in the fastigial nuclei [216]. In the latter, approximately one third of the neurons emit a burst of action potentials during saccades and modulate their firing rate during pursuit eye movements [217]. However, emitting a burst of action potentials does not necessarily imply that a neuron and its postsynaptic target belong to the saccade premotor network. The burst of these presumed “saccade–pursuit” neurons may be transmitted to precerebellar pursuit-related pontine nuclei and contribute to the postsaccadic enhancement of slow eye movements [218]. Finally, during unilateral inactivation of cFN, disorders in saccadic and pursuit eye movements are not correlated [107], whereas bilateral inactivation leads to bilateral hypermetria of saccades [92] while the gain of pursuit eye movements is either reduced [219] or unchanged [220].

In conclusion, behavioral observations provide helpful information for guiding neurophysiological investigations when they take into account anatomo-physiological constraints. Before giving several examples illustrating this benefit, we shall discuss the capacity to foresee the invisible and to “predict the future”.

### 3.3. About the “Predictive” Power of the Brain

Because they direct gaze toward locations that have not yet been crossed by the target, interceptive saccades have been called “predictive” and scenarios were proposed to explain this anticipation. For instance, the visuo-oculomotor system would be endowed with either a temporal countdown estimating “the time remaining until the collision of the target with the line of sight” [221] or a predictive clock announcing “the time at which the eye trajectory will cross the target” [222]. This conjecture led to imagining unrealistic options such as “a spatial lead of the gaze at the saccade end, instead of attempting a precise capture of the target” [221,223]. Indeed, the overshoot of saccades is incompatible with most published data. The majority of studies report that interceptive saccades either fall short or land approximately at the location where the target is at their landing time [159,160,161,162,163,164,224,225], even when their trajectory is perturbed by a change in eye position elicited by a brief electrical microstimulation in the SC [165]. Recent studies in monkeys showed that knowing the target path in advance does not lead to an overshoot of saccades either. Neither the accuracy nor the precision of saccades differs between blocks of trials in which the target path is always the same for one trial to the next and blocks in which the target path varies across the trials [164]. Saccades landing beyond the target location may occur in human subjects, but their occurrence is rare and conditioned by specific task instructions. They occasionally happen when the target moves at constant velocity on a periodic path. However, they are almost eliminated when the subject must accurately look at the target in order to detect a change in shape [226].

At the neuronal level, when a saccade is made toward a target moving at constant speed, saccade-related neurons in the deep SC emit a burst of action potentials as during saccades toward a static target. However, the population of active neurons does not contain commands related to future locations of a moving target. No action potentials are emitted by cells whose response field corresponds to upcoming saccadic vectors [227]. The active population consists of a continuum of cells ranging from neurons issuing commands related to past locations of the target to neurons issuing commands related to the current target location. Thus, the topography of active neurons does not change as fast as the target in the visual field because residual activities related to recently traveled locations persist.

The same conclusion holds for the results obtained in a preliminary study [228]. When comparing the response fields during saccades toward a static versus a moving target, a shift was found in the amplitude associated with the maximum firing rate. Cells exhibited a maximum discharge during saccades of larger amplitude when they were made toward a centrifugal target [see also 227]. However, in that study, the target moved so fast (60°/s) that it was possible that gaze did not capture it, unless the saccade was anticipatory. Indeed, a target that moves away from the central visual field with a speed of 60°/s after an initial jump of 2–6° reaches an eccentricity of 14–18° two hundred milliseconds later (approximate duration of response time). Since most measurements illustrated in this study’s representative figure [228] correspond to saccades smaller than 14 degrees, these could have been anticipatory, or their amplitude was strongly hypometric. If they were hypometric, then we do not understand why the putative additional command failed to compensate for the saccade undershoot. If the saccades were neither hypometric nor anticipatory, then the recorded bursts ought to correspond to that elicited by a past position of the target. Thus, the size of the shift in maximum firing rate between saccades to static and moving targets should be larger as the activity corresponds to positions further in the past. Current knowledge does not allow determining whether a horizontal diffusion of activity happens at the level of the SC or at the level of afferent input from structures of the cerebral cortex, such as area LIP [229,230] or FEF [160,231].

## 4. Neurophysiological Explanations

Conflating kinematic (or dynamic) numerical values with neurophysiological recordings carries the risk of believing that central neuron activity directly encodes gaze or head orientation rather than mediating changes in the contraction of extraocular and neck muscle fibers. Some studies examined how the firing rate of some central neurons could be accounted for by a combination of kinematic parameters such as current position, velocity and acceleration of the eyes [232,233,234], sometimes without separating the cells whose firing rate precedes the combination from those whose firing rate follows it [235,236]. Given (i) the spatial and temporal transformations that a brief spot of retinal excitation undergoes on its way to the motor neurons, (ii) the temporal overlap of activities corresponding to successive target positions, (iii) the multiple possible patterns of muscle contraction specifying any static orientation of gaze and (iv) the multiplicity of possible neuronal drives, identifying a neurophysiological correspondence of notions such as dynamic position error, velocity error or even velocity becomes challenging, otherwise vain (see also [34]).

Physical parameters are certainly convenient for interpreting and comparing the activity of neurons, sorting them into different groups and promoting scientific debates. However, the reservations made earlier about the block diagrams of oculomotor organization should not be restricted to the operations (differentiation, integration, gain, etc.) performed by the blocks [154,155]. They also concern the signals proposed to be “encoded” in the firing rate of neurons such as instantaneous motor error, velocity error or even velocity. The motor error command that feeds the premotor neurons during saccades toward a static target [207,237,238] might be less problematic because it is an oculocentric command [239] that can result from the preserved retinotopic arrangement of fibers connecting different neuronal assemblies.

To sum up, questioning the validity of a one-to-one correspondence relation between the sequence of values measured at the macroscopic level and the activity of microscopic neuronal elements does not lead the neurophysiology of gaze orientation to an epistemological dead end where the advancement of knowledge becomes vain or impossible. An alternative approach remains possible. Rather than reducing mismatches between extrinsic physical parameters (such as position error or velocity error), eye and head movements can be viewed as the behavioral expression of intrinsic processes that restore balances of activities opposing antagonistic visuomotor channels.

Interpreting the firing rate of neurons with notions belonging to the physical description of the movement of a body segment should be completed by the identification of muscle groups and neuronal networks involved. Comparative studies of the consequences of their functional perturbation and clinical and neuroanatomical studies should remain a privileged guide for interpreting neuronal activities and understanding their contribution to eye movement disorders [240,241]. Building upon the knowledge gathered during recent decades [242,243,244,245,246,247,248,249,250,251], the following sections explain how the poly-equilibrium is restored during eye saccades and during combined eye–head movements, while demonstrating the possibility of accounting for the neuronal basis of gaze visual orientation, without resorting to kinematic (or dynamic) notions.

### 4.1. Extraocular Muscles

**Figure 4:**
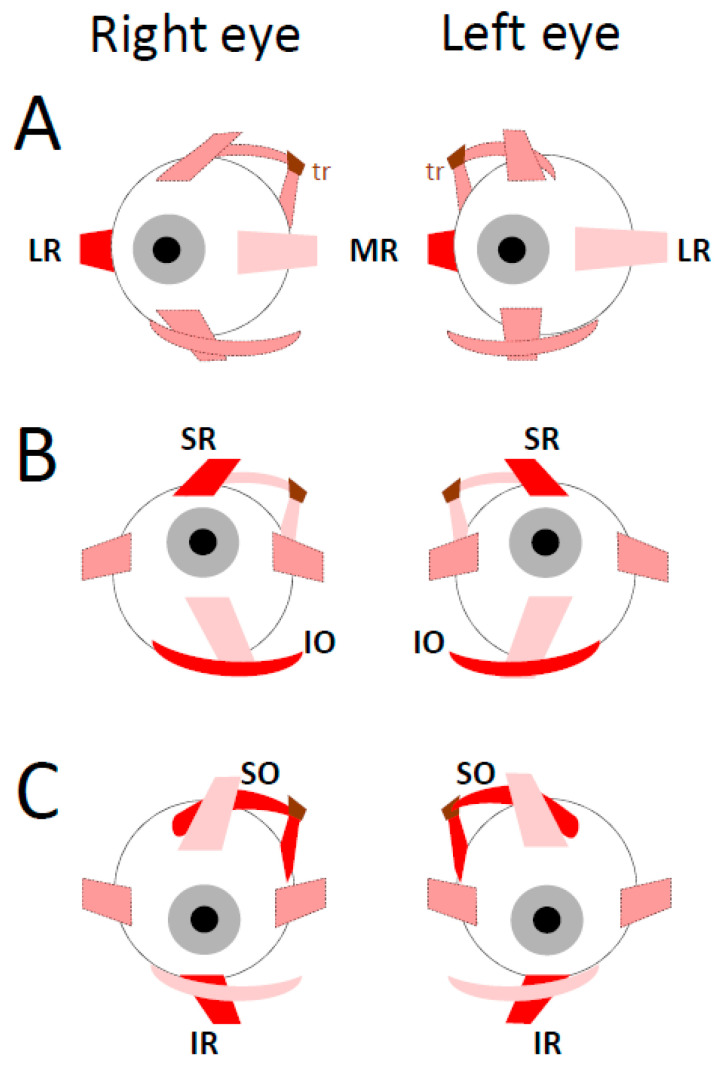
Schematic representation of extraocular muscles. LR: lateral rectus, MR: medial rectus, SR: superior rectus, IR: inferior rectus, SO: superior oblique, IO: inferior oblique, tr: trochlea. The muscles whose contraction rotates the eyes toward the right (**A**), upward (**B**) or downward (**C**) are colored in red. The muscles colored in pink are those that relax during the same rotations, respectively. The muscles outlined by a dashed black line sustain the same contraction level. Note that the SR and IR muscles are not positioned in planes running parallel to the midsagittal plane, as reported in [253,254,255,256].

Each eye movement involves synergies between anatomo-physiological elements that are invisible to investigations limited to behavioral analyses. However, the latter are helpful for discovering constraints in the contraction changes of the six muscles attached to the surface of each ocular globe (Figure 4). Combined with the relaxation of the medial rectus (MR) muscle fibers, the contraction of the lateral rectus (LR) muscle fibers of the same eye turns the eyeball toward the temporal side. Conversely, the contraction of MR combined with the relaxation of LR rotates the opposite eye toward the nasal side. In both cases, the eye rotates within the plane passing through the two muscles (toward the right in Figure 4A). These muscles are antagonistic to each other because their insertions are symmetrically positioned on opposite sides of the globe [252]. 

When the head is upright, the eye movement is horizontal provided that no change happens in the balances made by the contraction level of the other four extraocular muscles, i.e., between, on the one hand, the pair composed of superior rectus (SR) and inferior oblique (IO) muscles, and on the other hand, the pair composed of the inferior rectus (IR) and superior oblique (SO) muscles (Figure 4A). Any change in the balance of forces exerted by these two pairs will add a vertical component to the horizontal eye movement. Horizontal movements will exhibit a downward deflection if changes happen in the contraction of IR and SO muscle fibers (promoting depression) without concurrent changes in the contraction of SR and IO muscle fibers (promoting elevation). Reciprocally, horizontal movements will exhibit an upward deflection if changes happen in the contraction of SR and IO with no changes in the contraction of IR and SO muscle fibers.

An upward eye movement (elevation) results from the contraction of SR and IO muscle fibers of both eyes combined with the relaxation of IR and SO of both eyes (Figure 4B) whereas a downward eye movement (depression) results from the contraction of the latter combined with the relaxation of the former (Figure 4C). We shall see below that the trajectory of upward and downward saccades is straight vertical because of balanced input from neurons in the left and right caudal fastigial nuclei.

Anatomo-physiological studies indicated that, for each eye, distinct motor and premotor cells innervate these different muscles while behavioral studies report horizontal and vertical movements of both eyes with trajectories that are quasi-rectilinear (not curved) and torsionless. Visible to the external observer only (i.e., at the macroscopic level), such trajectories imply the existence of synergies in the recruitment of extraocular muscles, synergies that the sole anatomical survey cannot deduce easily. 

### 4.2. Binocular Synergy

Orienting movements of gaze toward a visual target involve both eyeballs. The rotation of the left eye results from changes in the contraction of the fibers of its extraocular muscles, which are innervated by motor neurons that are distinct from the cells innervating the muscles of the right eye. A synergy is thus required between the groups of premotor neurons for moving both eyes quasi-simultaneously. We shall see below the neural substrate of Hering’s law of equal innervation.

#### 4.2.1. Horizontal Eye Movements

**Figure 5:**
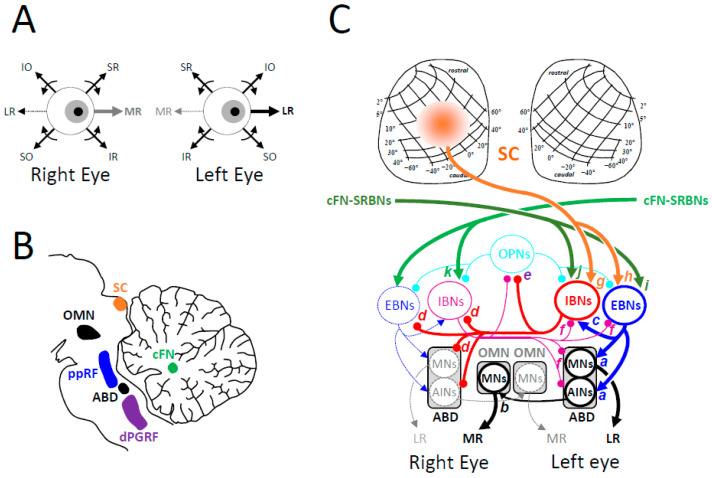
Neuronal network involved in the generation of leftward saccades. (**A**) The thickness of arrows attached to the eyeballs schematizes the strength of muscle contraction. LR: lateral rectus, MR: medial rectus, SR: superior rectus, IR: inferior rectus, SO: superior oblique, IO: inferior oblique. (**B**) Parasagittal section of the brainstem and cerebellum showing the approximate locations of the oculomotor nucleus (OMN), paramedian pontine reticular formation (ppRF), abducens nucleus (ABD), dorsal paragigantocellularis reticular formation (dPGRF), caudal fastigial nucleus (cFN) and superior colliculus (SC). (**C**) Connecting lines ended by an arrow indicate excitatory connections; those ended by a circle indicate inhibitory synaptic connections. The thickness of connecting lines schematizes the strength with which the neurons fire. SRBNs: saccade-related burst neurons, OPNs: omnipause neurons, EBNs: excitatory burst neurons, IBNs: inhibitory burst neurons, ABD: abducens nucleus, MNs: motoneurons, AINs: abducens internuclear neurons. Read text for explanations.

Figure 5 illustrates the network responsible for leftward rotations of both eyeballs, i.e., for the contraction of the left eye’s LR muscle and the right eye’s MR, combined with the relaxation of antagonist muscles (left eye’s MR muscle and right eye’s LR). As explained above, the contraction of the other four extraocular muscles (SO and IR against IO and SR) is maintained if the movement is straight horizontal (Figure 5A).

The degree of contraction of an extraocular muscle depends on the frequency of action potentials emitted by motor neurons innervating its fibers. For a horizontal eye movement, the motor cells innervating the LR muscle are grouped in the ipsilateral abducens nucleus whereas those innervating the MR muscle are in the ipsilateral oculomotor nucleus. The abducens nuclei are located in the pontine reticular formation and the oculomotor nuclei in the midbrain (Figure 5B).

The contraction of agonist muscles is caused by an increase in the firing rate of motor neurons (MNs) in the abducens nucleus for the LR muscle and in the oculomotor nucleus for the MR muscle (Figure 5C). During saccades, the muscle contraction is brisk not only because of the contractile properties of extraocular muscles [244,246,252] but also because the incoming input comes in a burst of action potentials with short interspike intervals [257,258,259,260,261,262]. In the abducens nucleus, internuclear neurons (AINs) relay to the motor neurons that innervate the MR muscle (synapse b) the excitation they receive at the same time as MN from excitatory burst neurons (EBNs, synapses a) located in the paramedian pontine reticular formation (ppRF) [150,263]. In human patients [176,177] and monkeys [264], a ppRF lesion eliminates the possibility of producing ipsilesional saccades (ipsilateral gaze palsy) whereas the generation of slow eye movements (during the vestibular ocular reflex or pursuit) remains possible. Electrical microstimulation applied to the ppRF evokes an ipsilateral movement of both eyes, which does not resemble a saccade. Its speed is most often constant and depends on the stimulation frequency [265,266]. The electrically evoked movement is not saccade-like for at least two reasons. First, the stimulation parameters (constant current and frequency of pulses) do not replicate the complete population burst that usually precedes each saccade. Indeed, a limitation of the electrical stimulation technique is that its strength cannot be enhanced without exciting unrelated fibers of passage and eliciting reactions other than oculomotor. Second, the antagonist muscles are not relaxed as they usually are during saccades. Their relaxation results from a pause in the firing rate of motor neurons, which is caused by action potentials emitted by inhibitory burst neurons (IBNs) located in the contralateral dorsal paragigantocellularis reticular formation (dPGRF) [212,267,268,269].

The consequences of dPGRF lesion have not yet been studied. However, because of the inhibition they exert on the motor neurons innervating the antagonist muscles (synapse d), the suppression of their burst is expected to hinder ipsilateral saccades and reduce their speed and amplitude. By disinhibiting IBNs located in the opposite side (synapse d), the unilateral suppression of IBNs’ activity is also expected to decrease the agonist drive during ipsilesional saccades (synapses f). By contrast, by disinhibiting EBNs and MNs on the opposite side (synapses d), the unilateral suppression of IBNs’ activity is also expected to increase the agonist drive during contralesional saccades and render them hypermetric. Recordings in monkeys reveal that approximately half of IBNs on the side opposite the recruited EBN emit action potentials during contralateral saccades [267,268,269]. These action potentials combine with those emitted by EBNs (synapses a and f) to compose the agonist oculomotor command [94,108,262,270,271].

Excitatory and inhibitory burst neurons receive descending inputs from saccade-related neurons in the contralateral SC (synapses g and h) and cFN (synapses i and j). The collicular input is monosynaptic in cats [272] but disynaptic in monkeys [273]. This difference between feline and primate species also concerns the fastigio-collicular projections. In monkeys, they are concentrated in the rostral part of both SCs [101] whereas they target more caudal regions in cats [274].

Finally, although premotor burst neurons receive lateralized input from the contralateral SC and cFN, the premotor control of saccades should be considered as bilateral [94,108,262,270,271] because of the omnidirectional bursts of saccade-related fastigial neurons. Indeed, for each horizontal saccade, a burst is emitted by neurons not only in the contralateral but also in the ipsilateral cFN [275,276,277,278]. The contralateral burst was proposed to accelerate the saccade by exciting the agonist drive from EBNs (synapses i and j) whereas the ipsilateral burst was supposed to decelerate or brake the saccade by recruiting IBNs on the opposite side (synapse k).

Several facts led to revision of this sequential activation and “biphasic” view of the fastigial control of saccade acceleration and deceleration [279] and the proposal of bilateral fastigial control of saccades [94,108,270,271]. First, the biphasic hypothesis is not applicable to the generation of vertical saccades [94,108]. Second, all IBNs do not fire during the late part of contralateral saccades; some of them burst at saccade onset [268,269], as EBNs do. The same remark holds for the burst of saccade-related fastigial neurons during ipsiversive saccades [278]. Moreover, the idea that the contralateral burst contributes to the acceleration of saccades is refuted by its later occurrence with larger amplitude of contralateral gaze shifts [280]. Yet, the maximum velocity of contralateral gaze shifts is significantly reduced during unilateral cFN inactivation [281]. Finally, still after unilateral cFN inactivation, ipsilateral saccades exhibit increased maximum velocity and enhanced displacement during the accelerating epoch, regardless of whether they are associated with a head movement or not [281]. Altogether, these observations are not compatible with the biphasic hypothesis but corroborate the bilateral hypothesis.

#### 4.2.2. Vertical Eye Movements

**Figure 6:**
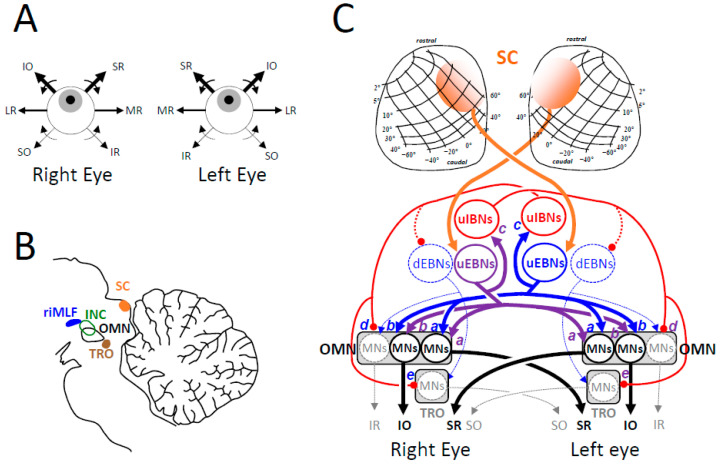
Network involved in the generation of upward saccades. (**A**) Same conventions as in Figure 5A. (**B**) Parasagittal section of the brainstem and cerebellum showing the approximate locations of the oculomotor nucleus (OMN), rostral interstitial nucleus of the medial longitudinal fasciculus (riMLF), interstitial nucleus of Cajal (iNC), trochlear nucleus (TRO) and superior colliculus (SC). (**C**) Connecting lines ended by an arrow and a circle indicate excitatory and inhibitory synaptic connections, respectively. The thickness of connecting lines schematizes the strength with which the neurons fire. Note the crossing axons of motor neurons innervating the SO and SR muscles. uEBNs: upward excitatory burst neurons, dEBNs: downward excitatory burst neurons, uIBNs: upward inhibitory burst neurons, MNs: motoneurons, SC: superior colliculus. Read text for explanations.

Contrary to the unilateral origin of the excitatory drive of horizontal saccades, the excitatory phasic drive is bilateral for saccades directed upward or downward (Figure 6). 

The contraction of agonist muscles is driven by a burst of action potentials emitted by motor neurons located in the ipsilateral and contralateral oculomotor nuclei (for the IO and SR muscle fibers, respectively) [282,283]. The relaxation of antagonist muscles results from a pause in the firing rate of motor neurons located in the ipsilateral oculomotor nucleus (for the IR muscles) and the contralateral trochlear nucleus (for the SO muscles) (Figure 6B). The agonist motor neurons receive a bilateral burst command from uEBNs located in both (left and right) rostral interstitial nuclei of the medial longitudinal fasciculus (riMLF; synapses a and b in Figure 6C) [284,285,286]. Likewise, the inhibition of antagonist motor neurons is bilateral and driven by uEBNs (synapses c). It originates from uIBNs located in the contralateral interstitial nuclei of Cajal (iNC; synapses d and e).

**Figure 7:**
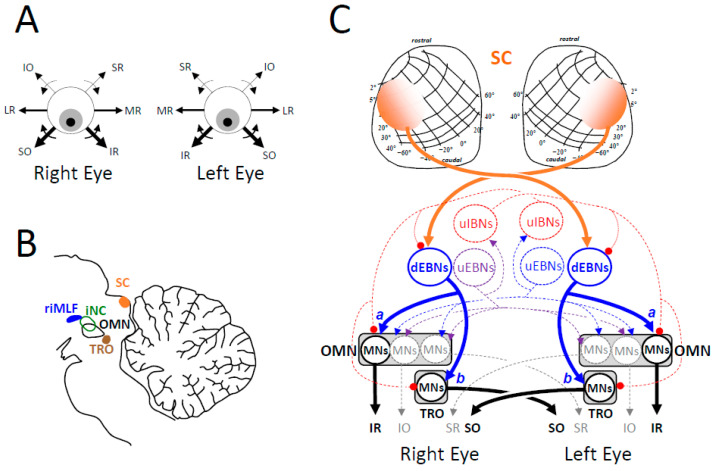
Network involved in the generation of downward saccades. Same conventions as in Figure 6. Read text for explanations.

The generation of downward saccade involves the contraction of IR and SO muscle fibers of both eyes combined with the binocular relaxation of SR and IO muscle fibers (Figure 7A). The contraction of agonist muscles is driven by a burst of action potentials emitted by motor neurons located in the ipsilateral oculomotor nucleus (for the IR muscles) and the contralateral trochlear nucleus (for the SO muscles) [287]. The relaxation of antagonist muscles results from a pause in the firing rate of motor neurons located in the ipsilateral oculomotor nucleus (for the IO muscles) and the contralateral one (for the SR muscles).

Contrary to the bilateral origin of the excitatory premotor drive of upward saccades, it seems to be unilateral for downward saccades [288], originating from dEBNs located in the ipsilateral riMLF (synapses a and b). The origin of the inhibition of antagonist motor neurons has not yet been identified [289]. The asymmetry of the excitatory input is consistent with the effects of unilateral lesions of riMLF since downward saccades are more impaired than upward ones [290].

It is worth remembering that (i) the contraction of SR and IO muscle fibers does not only elevate (supraduct) the eye during upward saccades and that (ii) the contraction of IR and SO muscle fibers does not only depress (infraduct) the eye during downward saccades. The contraction of SR and SO muscle fibers also causes intorsion (also called incycloduction) of the globe while the contraction of IO and IR muscle fibers causes extorsion (also called excycloduction). Thus, during upward saccades, the intorsion caused by the motor commands sent to the SR combines with the extorsion caused by the commands to the IO, whereas during downward saccades, the extorsion caused by activation of the IR muscle combines with the intorsion caused by activation of the SO muscle. Behavioral measurements made in healthy subjects reveal a negligible torsional component during vertical saccades [291], indicating that the intorsion and the extorsion cancel each other out.

During upward saccades, such a balance must result from bilateral activity in the midbrain because, for each eye, the motor neurons innervating the SR muscle are located in the contralateral OMN whereas the motor neurons innervating the IO muscle are located in the ipsilateral OMN (Figure 6C). Likewise, during downward saccades, the balance must also result from bilateral activity in the midbrain. The motor neurons innervating the IR muscle are located in the ipsilateral OMN whereas the motor neurons innervating the SO muscle are located in the contralateral trochlear nucleus (Figure 7C).

Thus, the generation of vertical saccades that are straight and torsionless rests upon adjusted bilateral motor activation, which must take into account both the structural (neurons and their connectivity) and functional (firing properties, secondary and tertiary consequences of muscle contractions) asymmetries existing between oculomotor territories distributed on either side of the brain stem. In monkeys, stimulation of the right riMLF produces a conjugate clockwise rotation of both eyes, whereas left riMLF stimulation produces counter clockwise rotation [292].

Since the generation of vertical saccades is bilateral, a torsional component should appear in vertical saccades after unilateral lesion of riMLF with a direction that depends upon the side of the lesion. When dEBNs in the right riMLF are lesioned, activity in the opposite (unimpaired) riMLF should provoke a binocular counterclockwise torsion, i.e., an intorsion of the right eye (caused by SO contraction) associated with an excyclotorsion of the left eye (caused by IR contraction). Clinical observations of a patient suffering from a unilateral midbrain lesion affecting the right riMLF confirm this oculomotor disorder. A counterclockwise torsional eye rotation accompanies each voluntary vertical saccade, downward and upward [293]. The fact that the patient’s upward saccades exhibited a counterclockwise torsion means that the saccade of the right eye (caused by SR contraction) was associated with an intorsion while the saccade of the left eye (caused by IO contraction) was associated with an extorsion.

These clinical observations are important because they suggest that the ipsilateral projection of uEBNs toward the oculomotor nucleus is stronger than the contralateral projection. Otherwise, during upward saccades, the counter clockwise torsion would have been counterbalanced by the clockwise torsion (resulting from the IO contraction in the right eye and from the SR contraction in the left eye) promoted by the crossed projections from uEBNs to the right OMN. More fundamentally, such inferences illustrate the mutual benefit of combining neurophysiological and neuroanatomical studies in non-human primates with clinical studies in patients for advancing our understanding of oculomotor disorders as well as normal physiology.

Finally, bilateral control of vertical saccades likely involves the two superior colliculi, with both medial halves driving upward saccades (Figure 6C) and both lateral halves driving downward saccades (Figure 7C). As mentioned earlier with the bilateral hypothesis, saccade-related neurons in the midline cerebellum may be involved in compensating for asymmetries and regulating the bilateral balance of activity between the left and right parts of the brainstem. Indeed, most neurons in the caudal fastigial nuclei [275,276] and in the vermis [294,295] emit a burst of action potentials during vertical saccades. The paucity of fastigial projections to the midbrain [296] and the observation that asymmetrical functional perturbation of the medio-posterior cerebellum primarily impairs the horizontal component of saccades [94] suggest a weak involvement in the generation of vertical and torsional components of saccades. Vertical saccades exhibit a deflection of their trajectory toward the side of caudal fastigial inactivation [94,108,297,298] or toward the opposite side when the vermis is asymmetrically lesioned [299,300].

However, further neuroanatomical studies should investigate the cerebellar connectivity with the midbrain because another clinical study reported a transient counterclockwise torsion during horizontal saccades in a patient suffering from a lesion involving the left deep cerebellar nuclei and the left lateral medulla oblongata [301]. Furthermore, future clinical studies should test whether bilaterally balanced lesions account for the rare changes in Listing’s law in patients suffering from cerebellar ataxia [302].

#### 4.2.3. Synergy of Horizontal and Vertical Saccades

The burst neurons involved in the generation of horizontal and vertical saccades are located in the pontomedullary reticular formation and the midbrain, respectively. Since the pontomedullary reticular formation is located more caudally than the midbrain, the distance “traveled” by a collicular action potential to postsynaptic premotor burst neurons differs between horizontal or vertical saccades. Yet, when we examine the velocity profiles of the horizontal and vertical components of oblique saccades, we see that the two components start at the same time. This synchrony may result from releasing the premotor burst neurons from the inhibition exerted by omnipause neurons [303,304]. Simultaneously released from their tonic inhibition (turquoise colored synapses in Figure 5), premotor burst neurons then can emit their burst of action potentials synchronously. Accordingly, lesions of RIP should desynchronize the onset of horizontal and vertical components during oblique saccades. No experiment has yet investigated this prediction.

Another coupling exists between the networks generating the horizontal and vertical components of oblique saccades. Its neurophysiological substrate has not been identified either. When the speed of one component diminishes, the other component is also slowed down. The duration of both components is lengthened during oblique saccades. When one compares cardinal and oblique saccades having equal horizontal displacement amplitudes, the horizontal component is slower during oblique saccades. Thus, the two components are not executed independently of each other. Their interaction, called component stretching, does not seem to originate only from the density of postsynaptic inputs from different collicular sectors to the populations of burst neurons in the ppRF and the midbrain [305]. A component stretching even occurs in oblique saccades evoked by an electrical microstimulation of the same collicular site, when the amplitude of one component is artificially varied by triggering the electrically evoked saccade at different times after a visual saccade [306].

The most spectacular demonstration of component stretching was shown after transient inactivation of neurons in the ppRF [307,308]. Therein, local injection of lidocaine reduced both horizontal and vertical components of oblique saccades. In some experiments, the inactivation was subtle enough to slow down the saccades without altering their accuracy. Consistent with the hypothesis of negative feedback control of saccade amplitude, the reported results also indicate that the elements that have been inactivated do not participate in the feedback that updates the residual motor error. They are also compatible with the hypothesis of poly-equilibrium insofar as saccade duration is prolonged if the activity of some neurons participating in its restoration is suppressed.

### 4.3. The Synergy of Eye and Head Movements

Orienting gaze toward the location of a stimulus often involves combined movements of the eyes and head. A change in the orientation of the trunk may also accompany the head movement [13]. When the body is at rest, with a head movement carrying the eyeballs, gaze movement amplitude corresponds to the sum of the amplitudes of the eye and head movements. Most of the time, gaze captures the target (i.e., its image is focused on the fovea) before the head stops moving because the saccadic eye movement is much faster than the head movement. Then, while the head continues to move, the eyes rotate in the opposite direction. The interval during which the eyes rotate in the direction opposite to the head movement is called vestibulo-ocular reflex. A slow counterrotation of the eyes in their orbit can also occur before the saccade, the head rotation preceding that of the eyes as during orienting toward an expected target location [309]. Thus, depending upon the amount of head rotation, the eye saccade will be of greater or lesser amplitude. The amplitude also depends on the orbital deviation of the eyeballs. If the eyes are deviated in the direction opposite to the direction of the impending saccade, the saccade amplitude is greater [12,310,311,312]; the eyes having a wider range of mobility before reaching the maximum deviation imposed by the oculomotor range. Consequently, the contribution of the head to the gaze displacement amplitude is smaller than during gaze movements initiated when the deviation of the eyes is closer to the limits of the oculomotor range. In the latter case, the head will start moving sooner. Thus, the contribution of the head to the gaze displacement amplitude depends also upon its onset relative to saccade onset. Regardless of whether the head moves or not, small gaze movements are equally accurate [312,313]. However, for eccentric targets requiring large gaze shifts, the accuracy is improved when a head movement accompanies the eye saccade [312].

In macaques, changing the orientation of the head involves at least twenty muscles [314]. Three groups can be distinguished: (i) the groups of muscles whose contraction changes the orientation of the head with respect to the trunk, (ii) the “orthogonal” groups of muscles whose contraction stabilizes the atlas while it rotates under the influence of the first group and (iii) the group of muscles that do not change their contraction level [315,316]. For instance, the co-contraction of the two recti capitis posterior minor (RCPm) muscles stabilizes the atlas while the contraction of each rectus capitis posterior major (RCPM) and obliquus capitis inferior (OCI) muscle produces ipsilateral horizontal rotation. The contralateral RCPM and OCI muscles, however, show no change in electromyographic (EMG) activity. This lack of change in the activity of antagonist muscles indicates that stopping the head rotation does not involve their contraction. For movements of amplitude greater than 20 degrees, the contraction of the splenius capitis muscle completes the contraction of RCPM and OCI muscles. The co-contraction of the RCPm, RCPM, OCI and obliquus capitis superior (OCS) muscles stabilizes the head at the end of its change in orientation.

In summary, when a head movement is involved, gaze-orienting response mobilizes several muscles, the synergy of which results from an activity involving a complex set of motor and premotor neurons, in the reticular formation, the vestibular nuclei and the spinal cord. In the continuation of studies performed in cats, neurophysiological investigations of eye–head movements in monkeys boomed in the 1990s and the beginning of the 21st century. They were dominated by the assumption that gaze direction was an intrinsic cerebral command involving saccade-related neurons in SC [88,89,136,317,318,319,320] and FEF [321]. This command would subsequently be fractionated into separate eye and head premotor commands, downstream of the SC (for alternative views, see [322,323]).

Three major obstacles hampered the development of neurophysiological investigations on eye–head movements. The first one was the technical difficulty of carrying out experiments with monkeys whose head is free to move. Added to this was the challenge posed by the necessity to merge new data collected in non-human primates with the considerable body of knowledge that had been obtained during prior experiments with cats. When differences between the feline and primate species were noticed (some of which have been reported in the text above), the relevance of data collected in non-primate animals to understand the physiology of the human brain was questioned. Third, the attractiveness of this developing research field in macaque monkeys was diminished by socio-political factors that promoted quickly published research works as well as the development of brain imaging and computer modeling studies. Faced with a migration of young investigators towards scientific fields in which the work was less risky, the neurophysiological study of eye–head movements in monkeys declined. Societal changes in European countries also complicated and rendered socially thankless the use of macaque monkeys for the advancement of scientific and neurological knowledge, discouraging the few researchers who remained engaged.

Fortunately, past results mostly obtained in cats left a treasure trove of data that allows a synthesis, which, although incomplete, illustrates the tremendous complexity that underlies the orienting movements of the eyes and head. The following text completes a recent report [131] with more detailed explanations and relevant references. It will serve as a guide for further investigations with marmosets or for comparison with other species.

#### 4.3.1. Reticulo-Vestibulo-Reticular Synergies

In cats, the recruitment of neck muscles during horizontal head movements exhibits some peculiarities. Although some muscles are systematically recruited, either phasically (splenius muscles, OCI, levator scapulae and complexus) or tonically (biventer cervicis), the recruitment of other muscles (semispinalis cervicis, longissimus, levator scapulae, scalenus anterior and OCS) depends on the orientation of the head relative to the trunk [324]. At the neurophysiological level, the motor network responsible for horizontal movements can be divided into three groups: an excitatory medial reticulospinal system and two medial vestibulospinal subsystems, a contralateral excitatory subsystem and an ipsilateral inhibitory subsystem. Contrary to macaques, almost every vestibulospinal neuron projects to the ocular motor nuclei in cats. In monkeys, the relative absence of cells carrying the same signals to motor neurons innervating the extraocular and neck muscles accounts for the dominance of the vestibulo-ocular reflex over the vestibulo-collic reflex [325].

##### The Reticulospinal Channel

Motor neurons innervating the agonist muscles receive signals from neurons located ipsilaterally in the rostrodorsal portion of the nucleus reticularis gigantocellularis (NRGC), the nucleus reticularis pontis caudalis (NRPC) and the dorsal portion of the nucleus reticularis gigantocellularis [326]. The combined lesion of the NRPC and NRGC abolishes the generation of ipsilateral head movements almost completely [327]. Ipsilesional saccadic eye movements are then also absent. However, contralateral saccades and vertical movements of the eyes and head do not seem to be affected. The slow compensatory movements of the vestibulo-ocular and vestibulo-collic reflexes are also spared.

The firing properties and connectivity of reticulospinal neurons are compatible with their involvement in the generation of combined ipsilateral eye and head movements [328]. Indeed, eye–neck reticulospinal neurons (EN-RSNs) emit bursts of action potentials during ipsilateral eye saccades associated with ipsilateral EMG activity of neck muscles. When the eyes are deviated toward the contralateral side and the ipsilateral neck muscles relaxed, the neurons are silent. Action potentials are emitted only when the saccade leads the eyes beyond the orbital sagittal plane. None is emitted when the saccade is made in the contralateral orbital hemifield. This sensitivity to the orbital eye deviation, which appears neither in tecto-reticulo-spinal neurons [329,330] nor in cortico-reticulo-spinal neurons [331], may result from afferents from neurons in the nucleus prepositus hypoglossi (NPH). Therein, neurons display a sustained firing rate that increases with ipsilateral deviation of the eyes in the orbit. When the eyes are deviated toward the contralateral side, they are silent [332]. Thus, NPH neurons may account for silencing the EN-RSNs because their projections are primarily contralateral and inhibitory.

In cats, a projection of OPNs onto the EN-RSNs has been interpreted as evidence for an involvement of OPNs not only in the generation of eye saccades but also in eye–head gaze saccades [333]. This idea is compatible with a correlation between the duration of their pause and gaze saccade duration stronger than the correlation between the pause duration and the duration of eye saccades [334]. It is also compatible with the slowing of head movement when a short electrical microstimulation is applied in the RIP nucleus [334]. However, the alternative interpretation that OPNs remain primarily involved in the generation of ocular saccades cannot be rejected. The fact that the eye saccade duration is shorter than the gaze saccade duration does not necessarily imply that the burst duration of premotor neurons is also shorter. The shorter duration of eye saccades may result from the action of the vestibulo-ocular reflex downstream, at the level of motor neurons. Thus, the correlation between the pause duration of OPNs and the duration of eye saccades would be less strong than the correlation with the duration of gaze saccades. The slowing of head movement by electrical microstimulation of RIP may result from a transient reactivation of the vestibulo-collic and vestibulo-ocular reflexes because of the inhibition that OPNs exert upon excitatory and inhibitory burst neurons [91,303] (turquoise colored synapses in Figure 5). Finally, it is worth signaling that, in monkeys, (i) a slowing of head movement is rarely observed when RIP is stimulated [27] and (ii) the end of the OPN’s pause is better correlated with the eye saccade end than with the gaze shift end [335].

##### The Vestibulospinal Channels

The medial vestibulospinal tract (MVST) is the major pathway by which afferent signals from the horizontal semicircular canals influence the axial musculature of a cat’s body. A trisynaptic pathway connects the labyrinth to the motor neurons innervating the neck muscles [336,337]. The neurons at the origin of the vestibulo-nucal pathway are secondary vestibular neurons located in the medial vestibular nucleus (MVN). Activated by ipsilateral vestibular afferents, the firing rate of these neurons increases during an ipsilateral rotation of the head and decreases during a contralateral rotation, a response pattern called type I [338,339]. The postsynaptic influence of the MVST on the motor neurons of the contralateral cervical cord is excitatory. An inhibitory monosynaptic projection from the MVN to ipsilateral neck motoneurons has also been shown [336,338,340]. The fact that a stimulation of the horizontal canal evokes a head movement directed towards the contralateral side [341] is compatible with this organization. Many neurons of the MVST, which are mainly located in the rostral MVN, emit a collateral towards the contralateral abducens nucleus [342,343,344]. This double connection with the extraocular and head motor neurons demonstrates their involvement in combined eye–head movements.

Electrophysiological studies report high sustained activity of eye–neck vestibular neurons when the eyes are centered in the orbit [345]. This tonic firing rate results either from an intrinsic property of neurons or from their sustained excitation by primary vestibular neurons [346]. In some vestibular neurons, a sensitivity to the deviation of the head relative to the trunk suggests an influence also from neck proprioceptive signals [347].

During the passive rotation of the head, the vestibular signals inhibit the motor neurons innervating the ipsilateral extraocular and neck muscles while facilitating the activity of those innervating the contralateral muscles. Thus, the rotation of the head promotes the emission of vestibular signals, which leads to the counterrotation of the eyes (vestibulo-ocular reflex) and of the head (vestibulo-collic reflex) in the direction opposite to the head rotation. These reflex movements also oppose active rotations of the head. We shall now examine how saccade-related commands remove this opposition.

#### 4.3.2. Inhibition of Vestibulo-Ocular and Vestibulo-Collic Reflexes

**Figure 8:**
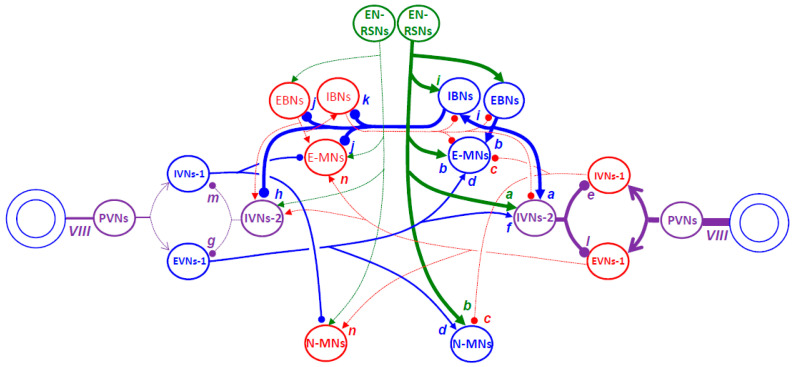
Neuronal network involved in combined horizontal eye and head movements. Connecting lines ended by an arrow indicate excitatory connections; those ended by a circle indicate inhibitory synaptic connections. Blue color indicates the agonist neuronal oculomotor elements, red color the antagonist ones. Green color indicates the cephalomotor elements. The thickness of connecting lines schematizes the strength with which the neurons fire. EN-RSNs: eye–neck reticulospinal neurons, EBNs: excitatory burst neurons, IBNs: inhibitory burst neurons, E-MNs: abducens motor and internuclear neurons, N-MNs: neck motoneurons, PVNs: primary vestibular neurons, IVNs: inhibitory vestibular neurons, EVNs: excitatory vestibular neurons, VIII: eighth cranial nerve. Read text for explanations.

In addition to excitatory inputs from EBNs and EN-RSNs (synapses b in Figure 8), the motor neurons innervating the agonist eye and neck muscles (E-MNs and N-MNs, respectively) also receive inhibitory afferents from the IVNs-1 located in the ipsilateral MVN (synapses c) and excitatory afferents from the EVNs-1 in the contralateral MVN (synapses d). The inhibition of agonist motor neurons (by IVNs-1, synapse c) is prevented by action potentials emitted by IVNs-2 (synapse e), which are recruited by EN-RSNs and EBNs (synapses a) and by action potentials emitted by EVNs-1 located in the contralateral MVN (synapses f). These contralateral EVNs-1 are indeed released from the inhibition of IVNs-2 (synapse g) by the burst of action potentials emitted by IBNs (synapse h), which are recruited by agonist premotor neurons (synapses i). Thus, a disinhibition facilitates the recruitment of agonist muscles while the relaxation of antagonist muscles is promoted by the inhibition that IBNs exert on IVNs-2 in the contralateral MVN (synapse h) and on antagonist ocular premotor and motor neurons (synapses j).

During a combined horizontal movement of the eyes and head, the eyes do not counterrotate in their orbit (vestibulo-ocular reflex) because the motor and internuclear neurons (E-MNs) in the contralateral abducens nucleus are inhibited by IBNs (synapse j). By exciting the IVNs-2 (synapses a), the bursts emitted by RSNs and EBNs prevent the ipsilateral IVNs-1 from thwarting the recruitment of agonist motor neurons (synapses c). The counterrotation of the eyes restarts as the firing rate of ipsilateral EBNs and IBNs diminishes. The declining firing rate of EBNs removes the inhibition that ipsilateral IVNs-2 exert upon IVNs-1 and EVNs-1 (synapses e and l) while the declining firing rate of IBNs disinhibits IVNs-2 on the contralateral side (synapse h), which start inhibiting the EVNs-1 and IVNs-2 (synapses g and m). The ipsilateral MNs are inhibited by the IVNs-1 (synapse c) while the contralateral MNs can again emit action potentials in proportion to the excitation they receive from the contralateral EVNs-1 (synapses n) while the head movement terminates. Since this excitation recruits the antagonist neck muscles, it may contribute to stopping the head movement [348,349]. However, it is worth reminding that in monkeys, this co-contraction has been questioned by EMG studies [315,316].

In summary, the firing rate reduction of IVNs-2 contributes to ending the ipsilateral orienting gaze shift by two parallel processes. The first process is the consequence of the gradual decline in excitation from EBNs and EN-RSNs, while the second process results from an inhibition by IBNs on the contralateral side. In monkeys, the decline in the firing rate of EBNs and EN-RSNs might not result from a reduced drive from collicular neurons because the latter often continue to fire after saccade end [66,350,351,352,353,354]. Instead of a reduced drive, a desynchronization of presynaptic input to premotor neurons may happen. As explained repeatedly in the text above and elsewhere [52], temporally scattered presynaptic inputs are less efficient than simultaneous ones for exciting postsynaptic neurons. The decline in EBN and EN-RSN activity may result from the inhibitory input from IBNs on the contralateral side. Thus, action potentials emitted by IBNs during contralateral saccades would participate in the inhibition of contralateral IVNs-2 and promote the counterrotation of the eyes. This cascade of events could account for the hypometria of contralesional saccades and the “premature” triggering of vestibulo-ocular reflex, making gaze shifts hypometric after unilateral inactivation of cFN in monkeys [281].

## 5. Conclusions

Orienting gaze movements enable an animal to localize and capture an object in its physical environment. They are convenient means for investigating how networks of neurons govern oculomotor behavior [355] and underlie behavioral performances, notably when neurological damage alters their normal functioning [240,241]. During recent decades, following the steps of predecessors who conducted their research in feline species, neurophysiologists and neuroanatomists gathered considerable knowledge in monkeys that contributed to identifying the core networks involved in the generation of eye and head movements and to understanding the oculomotor disorders of human patients.

Technological advancements brought the possibility to measure precisely the time course of eye and head movements and to study correlations between the firing rate of neurons and various kinematic and dynamic parameters (amplitude, velocity and acceleration) or the angular distance between gaze and target directions. Analytical habits, which became conventions, resulted and gradually led some neuroscientists to assume a one-to-one correspondence relation between neuronal activity and measured physical values of gaze or head orientation. From there, the search for neurophysiological evidence supporting their theoretical diagram was promoted.

However, comparing kinematic or dynamic numerical values with neurophysiological recordings carries the risk of believing that central neuron activity directly encodes gaze or head orientation rather than mediating changes in extraocular and neck muscle contraction, in posture, in the autonomous nervous system and more centrally. Neuroanatomical and neurophysiological studies indeed reveal functional properties and connectivity that are not visible in cybernetic diagrams. The comparison between a diagram and the brain is analogous to that between an automaton and a living animal.

Complementing a previous synthesis [131], this review article is enriched by epistemological comments on the notion of “space” in the brain and by numerous additional precisions and references. It also provides a solid ground of evidence for a new departure in the neurophysiological study of orienting movements by considering static gaze orientation as poly-equilibrium, i.e., multiple balances of neuronal activities opposing mutually antagonistic channels. A saccade or a slow eye movement is not initiated as long as the visuo-oculomotor system is within in a mode where opposing commands counterbalance each other. For generating saccadic and pursuit eye movements, the symmetry breaking involves different groups of neurons in the left and right parts of the brainstem. Thus, rather than reducing position or error signals—which are numerical values belonging to the physical domain of kinematics—orienting movements of the eyes and head are the behavioral expressions of intrinsic neuronal processes restoring the poly-equilibrium. Further investigations will connect them with processes involved in postural adjustments as well as with those that do not belong to classical visuomotor functions, but to autonomous functions [356,357] and alertness [358,359].

## Data Availability

No new data were created or analyzed in this study.

## Abbreviations

ABD	abducens nucleus
AIN	abducens internuclear neuron
cFN	caudal fastigial nucleus
dPGRF	dorsal paragigantocellularis reticular formation
EBN	excitatory burst neuron
E-MN	eye motoneuron
EVN-1(2)	excitatory vestibular neuron of type 1 (type 2)
FEF	frontal eye field
IBN	inhibitory burst neuron
iNC	interstitial nucleus of Cajal
IO	inferior oblique
IR	inferior rectus
IVN-1(2)	inhibitory vestibular neuron of type 1 (type 2)
LR	lateral rectus
MLF	medial longitudinal fasciculus
MN	motoneuron
MR	medial rectus
MVN	medial vestibular nucleus
MVST	medial vestibulospinal tract
N-MN	neck motoneuron
NOT	nucleus of the optic tract
NPH	nucleus prepositus hypoglossi
NRGC	nucleus reticularis gigantocellularis
NRPC	nucleus reticularis pontis caudalis
OMN	oculomotor nucleus
ppRF	paramedian pontine reticular formation
RIP	raphe interpositus
riMLF	rostral intersitial nucleus of MLF
EN-RSN	eye–neck reticulospinal neuron
SC	superior colliculus
SO	superior oblique
TRO	trochlear nucleus
